# Strategies to address non-communicable diseases in the Commonwealth of Independent States countries: a scoping review

**DOI:** 10.1017/S1463423622000639

**Published:** 2022-11-15

**Authors:** Elvira Muratalieva, Mathieu Nendaz, David Beran

**Affiliations:** 1 Biomedical Sciences Global Health Track at the University of Geneva, Genève, Switzerland; 2 Swiss Development and Cooperation in the Kyrgyz Republic and Embassy of Switzerland in the Kyrgyz Republic, 21 Erkindik boulevard, Bishkek 720040, Kyrgyzstan; 3 Medical Education Development and Research Unit (UDREM), Department of General Internal Medicine, Faculty of Medicine, Université de Genève, 1211 Genève 4, Switzerland; 4 Division of Tropical and Humanitarian Medicine, Geneva University Hospitals and Université de Genève, 1211 Genève 4, Switzerland

**Keywords:** countries CIS, diabetes, hypertension, non-communicable diseases, PEN protocol, Primary HealthCare system

## Abstract

**Aim:**

The aim of this study is to review the literature in Commonwealth of Independent States (CIS) countries with regard to their response to non-communicable diseases (NCDs) and the implementation of the World Health Organization (WHO) Package of Essential Non-communicable (PEN) disease interventions for primary health care.

**Background:**

NCDs are estimated to account from 62% to 92% of total deaths in CIS countries. Current management of NCDs in CIS countries is focused on specialists and hospital care versus primary health care (PHC) as recommended by the WHO.

**Methods:**

This paper uses a scoping review of published and grey literature focusing on diabetes and hypertension in CIS countries. These two conditions are chosen as they represent a large burden in CIS countries and are included in the responses proposed by the WHO PEN.

**Findings:**

A total of 96 documents were identified and analysed with the results presented using the WHO Health System Building Blocks. Most of the publications identified focused on the service delivery (41) and human resources (20) components, while few addressed information and research (17), and only one publication was related to medical products. As for their disease of focus, most studies focused on hypertension (14) and much less on diabetes (3). The most studies came from Russia (18), followed by Ukraine (21) and then Kazakhstan (12). Only two countries Moldova and Kyrgyzstan have piloted the WHO PEN. Overall, the studies identified highlight the importance of the PHC system to better control and manage NCDs in CIS countries. However, these present only strategies versus concrete interventions. One of the main challenges is that NCD care at PHC in CIS countries continues to be predominantly provided by specialists in addition to focusing on treatment versus preventative services.

## Background

Non-communicable diseases (NCDs) kill 41 million people each year, equivalent to 71% of all deaths globally (WHO, 2022). Each year, 15 million people die from an NCD between the ages of 30 and 69 with over 85% of these ‘premature’ deaths occurring in low- and middle-income countries (LMIC) (Martinez *et al.*, 2020). Among the NCDs, cardiovascular diseases account for most deaths, with 17.9 million people annually, followed by cancers (9.0 million), respiratory diseases (3.9 million), and diabetes (1.6 million). To address this challenge, the World Health Organization (WHO) has elaborated and introduced the 2008–2013 Action Plan for the Global Strategy for the Prevention and Control of NCDs (WHO, 2008). The Plan provides a roadmap for addressing NCDs at the country and global levels by strengthening surveillance; taking action to reduce risk factors with emphasis on interventions that are affordable and known to work; and addressing gaps in health systems and improving access to essential health care for people with NCDs. In addition to this multisectoral plan, WHO has elaborated a conceptual framework, the Package of Essential Non-communicable (PEN) disease interventions for primary healthcare (PHC) system in low-resource settings. This instrument was developed to strengthen equity and efficiency at PHC and defines a minimum set of cost-effective interventions, which include methods for early detection and prevention of NCDs.

In the Commonwealth of Independent States (CIS) (Armenia, Azerbaijan, Belarus, Georgia, Kazakhstan, Kyrgyzstan, Moldova, Russia, Tajikistan, Turkmenistan, Ukraine and Uzbekistan), NCDs are estimated to account from 62% to 92% of total deaths (WHO, 2014). When CIS countries were part of the Union of Soviet Socialistic Republic (USSR), the health system, Semashko system, was characterised by a centralised (state-run model) healthcare system with strong emphasis on specialists and hospital care (Balabanova *et al*., 2012). The Soviet Polyclinics (outpatient facilities) were represented by narrow specialists only, while paediatricians and ‘therapists’ were considered as generalists for children and adults. However, the gatekeeping function of PHC system was not in place. Specialists managed individual diseases, and this resulted in vertical systems, such as cardiology, endocrinology, gastroenterology. In the case of multi-morbidity, people were cared for by 2–3 specialists. At the same time, the Semashko model also considered prevention, which was called ‘dispensarisation’ and included massive screening and check-ups for specific diseases. From health financing point of view, health care was free for the patients, but very expensive for the Government of the USSR (Birn and Krementsov, 2018). Therefore, after collapse of the USSR, the Soviet Republics were unable to maintain this model which resulted in a crisis for the newly independent ex-Soviet states in the delivery of health care (Balabanova *et al*., 2012).

The epidemiological situation for NCDs in CIS countries requires PHC to play a strong role in the prevention and management of these conditions (Barbazza *et al*., 2019). However, despite the cost-effectiveness of PHC and evidence that exists on the affordability of preventive measures and early detection of NCDs at this level of care (Boitsov and Vylegzhanin, 2015) gaps exist at the PHC level in CIS countries (Kasimova, 2018). Therefore, the aim of this review is to identify how CIS countries have responded to the NCD burden and the implemented the WHO PEN.

## Methods

This paper uses a scoping review of published and grey literature as proposed by Arksay and O’Malley (O’Malley, 2002). This paper uses a focus on diabetes and hypertension in CIS countries. These two conditions are chosen as they represent a large burden in CIS countries and are included in the responses proposed by the WHO PEN. Scoping reviews include four subsequent stages: identifying the research question, identifying relevant studies, study selection, summarising, and reporting the results.

The research question for this study was: What is known from the existing literature about hypertension and diabetes management within PHC in the CIS countries? The search was conducted using the following sources: PubMed, WHO web page (Health topic NCDs) and the Russian scientific electronic library eLIBRARY.RU. The following keywords were used in the search strategy: Primary Healthcare system, non-communicable diseases, hypertension, diabetes, PEN protocol, countries CIS: all 12 (Russia, Belorussia, Ukraine, Moldova, Armenia, Azerbaijan, Georgia, Kazakhstan, Kyrgyzstan, Tajikistan, Turkmenistan, Uzbekistan). Peer-reviewed and grey literature in English and Russian from 2010 to 2020 were included. During the review, the identified publications were assessed for eligibility against the following inclusion criteria:

Study takes place in a CIS country

Studies that describe hypertension and diabetes management and control

Publication includes the role of PHC

The results of the literature review were analysed by a single person using the WHO Health System Building Blocks (WHO, 2010):

1. Leadership/governance
2. Healthcare Financing
3. Health Workforce
4. Medical products, technologies
5. Information and research
6. Service delivery

## Results of the scoping review

**Figure 1.**
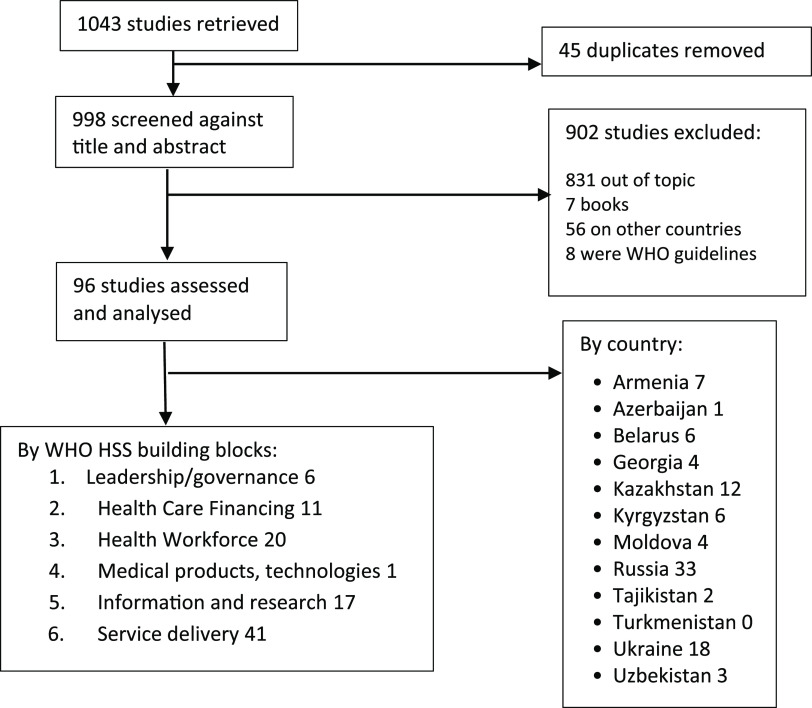
Summary of the publications selection in the study.

In total, of the 1,043 studies identified 96 publications were included in this study (see in Figure 1).

### 1. Leadership/governance

WHO determined that effective leadership and governance are one of the health system building blocks, which are focused on the policies that allows the health system to function and deliver services (WHO, 2010). These policies can be around disease-related policies or cross-cutting policy areas. Thus for this element, it is important to look at both policies regarding NCDs and PHC.

Under this building block, six publications were identified and reviewed. One group of publications focused on the implementation of the WHO’ Global Action Plan for the prevention and control of NCDs for the period of 2013–2020. Following this action plan, the CIS countries developed their own national NCD action plans with strong advocacy from WHO (WHO-Moldova, 2018; WHO-Ukraine, 2018). For example, Ukraine’s NCD action plan was designed with three main objectives: intersectoral action and partnership around NCD risk factors, prevention of NCDs in clinical and community settings, and reduction of risky behaviours influencing NCD mortality and morbidity (WHO-Ukraine, 2018). The NCD action plan of Moldova was similar to the one in Ukraine with additional plans focusing on alcohol control (WHO-Moldova, 2018).

The overall assessment of the NCD Action plan was done by WHO in Ukraine (WHO-Ukraine, 2018) and Moldova (WHO-Moldova, 2018) with these countries having elaborated several strategies around the NCD-related risk factors, but their enforcement was not assessed and documented. NCD management and control are considered as multisectoral task, and the countries developed certain intersectoral objectives. However, most of these actions remained on paper and did not materialise due to difficulties of multisectoral work.

Concerning PHC and governance in CIS countries, WHO has evaluated and reported on the PEN piloting in Moldova and Kyrgyzstan (Jill Farrington, 2017; Collins *et al*., 2019). The results of these evaluations were not very promising due to the short period of piloting during one year. In one year, the NCD management practices were changed in the PHC facilities, but time was insufficient to demonstrate improvement of health outcomes. In March 2017, WHO conducted a workshop on implementation of PEN in Eastern Europe and Central Asia (WHO-Europe, 2017), which stated that out of 12 CIS countries only five of them had piloted the PEN protocol. From the literature review, it was found that none of the CIS countries had their own vision or strategy to strengthen PHC (WHO-Europe, 2017). This is despite the fact that two global conferences on PHC were conducted in Kazakhstan one of the CIS countries (Exworthy, 2008).

### 2. Healthcare financing

Health financing requires the system to raise sufficient resources to provide health care in parallel to protecting the population from the financial burden of paying for health services (WHO, 2010).

Eleven publications were identified that address health financing. The studies from CIS countries show that there is no clear health financing reform towards strengthening the PHC system (Rechel *et al.*, 2013). Data from CIS countries describe the overall cost-effectiveness of PHC (Balashova *et al.*, 2014) as well as for preventive measures and early detection of NCDs in Ukraine (Palamar and Gruzeva, 2019).

CIS countries still prioritise hospitals versus PHC, with, for example, Russia spending more on inpatient care than outpatient: 50.3% and 33.2%, respectively (Alshamsan *et al.*, 2017; Sheiman *et al.*, 2018). Also, development assistance is very limited (US$ 0.34 per capita in CEE-CIS countries) and fragmented for PHC strengthening in CIS countries (Suhrcke et al., 2005).

The main approach applied for PHC financing is per capita payment, which is not covering all required services (Smith, 2011; Mukeeva, 2012; Schwarz *et al.*, 2013). The capitation-based financing of PHC in CIS countries is very low and does not exceed 35% of overall government health expenditure (Oleszczyk *et al.*, 2012; Shvets, 2012). Therefore, the PHC system in CIS countries is considerably underfinanced, which increases out-of-pocket payments for the population (Rechel *et al.*, 2013; Schwarz *et al.,* 2013; John Tayu Lee *et al*., 2015) mainly with regard to diagnostic and laboratory tests and medicines.

One study highlighted how in Kazakhstan the Government’s financing for PHC was increased in 2014 from USD 1.69 billion to USD 2.61 billion, but did not improve the gatekeeping and prevention functions of PHC (Birtanov, 2016). Other studies show that the salaries of the PHC workers are fixed salaries without incentives based on performance (Mukeeva, 2012). This results in low motivation of doctors and nurses to work more and cover health promotion and counselling on NCD-related risk factors and might explain why additional funding without addressing human resource issues results in little change.

### 3. Health workforce

A key component of any health system is the health workforce delivering care and services to the population.

In 1978, the Alma-Ata declaration on PHC was adopted by the USSR together with other countries and was considered as an action towards structural reforms from the Soviet policlinics to PHC centres (Exworthy, 2008). The reorganisation of policlinics to the primary healthcare centres was declared in 1990 in Russia, but without clear vision or strategy to strengthen PHC. This resulted in functioning of Soviet policlinics as they used to work before. One of the main reasons was the absence of comprehensive training for general practitioners. A study in 2016 shows that only 20% of the universities had general practice as a subject in pregraduate medical education (Gruzeva *et al*., 2016a, 2016b). Medical education systems in CIS countries were continuously training specialists instead of generalists (Parfitt, 2009; Mukeeva, 2012; Brimkulov, 2015). This resulted in the PHC system that mainly functioning as an outpatient facility where healthcare services are provided by specialists.

During the last five years, in some CIS countries the health system has tried to introduce family medicine practice by retraining specialists (Barton, 2011; Kolesnyk and Svab, 2013; Voronenko and Shekera, 2013; Alikhanova *et al.*, 2017). This resulted in gradual increasing family doctors, but with very low competence. A study conducted in 5 CIS countries to evaluate the quality of care through the clinical performance and value vignettes (CPV) demonstrated very low knowledge of the general practitioners about NCD management and its risk factors (Hrytsko *et al*., 2014; Peabody *et al.*, 2017; Matiukha *et al.*, 2020). Coexistence of specialists in PHC facilities does not motivate family doctors to learn more, because it is easier to refer them to specialists (Asadov and Aripov, 2009; Oleszczyk *et al.*, 2012). Therefore, it is important to prepare health professionals for PHC with a focus on prevention and early detection of diseases at undergraduate, postgraduate, and continuous medical education levels (Lilit Khachatryan, 2013; Krzton-Krolewiecka *et al*., 2013; Gruzeva *et al*., 2016a, 2016b). A study conducted in 30 European countries and some CIS countries confirmed that building General Practice/Family Medicine in academic discipline comprising teaching and research was essential to strengthen the PHC system (Zarbailov *et al.*, 2017; Kasimova, 2018; Akhmedov and Jarylkasynova, 2010). However, the interest of students to become general practitioners in Russia was ≤5% in 2009 (*Krzton-Krolewiecka et al.*, 2013). This situation is even worsened by globalisation when migration is increasingly present among medical personnel.

### 4. Medical products, technologies

Medicines and supplies need to be provided within the health system and are an essential pillar of the health system. Only one publication was identified as relevant to this building block.

This study aimed to assess the availability, prices, and affordability of four essential medicines used to treat diabetes in private primary care pharmacies in seventeen countries including Russia (Babar *et al.*, 2019). From this study, availability of some diabetes medicines was poor, but affordability was better than in the other countries included in this study.

### 5. Information and research

Another building block proposed by the WHO is the information and research that is produced by the health system. In the literature review, seventeen publications described the information and research building block.

These articles identified for this building block can be divided into two groups: firstly, articles describing the intentions of CIS countries to develop family medicine (Asadov and Aripov, 2009; Akhmedov and Jarylkasynova, 2010; Balabanova *et al*., 2012; Peabody *et al.*, 2017; Blake *et al.*, 2019). In most of these articles, authors are documenting shift from soviet system of polyclinics to family medicine/general practice without appropriate financing and human resources reforms (Gazizov, 2010; Balabanova *et al*., 2012).

The second group of articles are studies done by academic institutions on the management of chronic diseases at the PHC facilities and NCD burden in different CIS countries (Gazizov, 2010; Ryngach and Vlasyk, 2018; Blanche Greene-Cramer *et al*. 2020). These include studies on management of hypertension and diabetes at the PHC level (Lysenko, 2011; Codreanu *et al.*, 2012; Ahmedov *et al*., 2013; Basu and Millett, 2013; Chizova and Oshchepkova, 2013; Shinbolatova *et al.*, 2014; Zhdan *et al.*, 2017; Bikbov *et al*., 2019). Many of these studies confirm the effectiveness and affordability of the PHC system in the management of chronic diseases (Ahmedov *et al*., 2013; Barbazza *et al.*, 2019; Collins *et al.*, 2019). One of the main reasons of effectiveness of the PHC system reported in these studies is the focus on prevention of NCD-related risk factors (Rahmonov et al., 2012; Avdeeva, 2013; Polyanskaya, 2013; Palamar and Gruzeva, 2018; Gruzieva et al., 2018).

### 6. Service delivery

Service delivery is the health system providing quality, accessible, and safe services to the population. In the reviewed literature, 41 identified documents discuss this component of the health system.

PHC in the most CIS countries is disease-centric and does not include a patient-centred approaches (Birn and Krementsov, 2018; Beran *et al.*, 2019). Prevention-centric approaches are missing in PHC in all CIS countries (Luck *et al.*, 2014). The state system is still promoting disease management at PHC and forgetting the key role of disease prevention and early detection, which is an essential component of PHC (Dzhakeli *et al.*, 2009; Efremov, 2011; Sharman, 2014; Mirzikashvili and Baramidze, 2017). A study on the NCD-related risk factors conducted in 27 countries, including CIS countries, confirmed that addressing the risk factors plays a key role in preventing most of NCDs (Kotseva, 2019). At the same time, most of the services at PHC are provided by specialists (Konysbaeva, 2017) and very little by family doctors (Boitsov and Vylegzhanin, 2015; Boitsov and Fleck, 2015; Smiianov *et al.*, 2018; Kononov *et al.*, 2019). In some countries, some specialists (therapist, gynaecologist, and paediatrician) were retrained as family group practitioners to replace specialist-based care with family medicine approach (Verulava *et al.*, 2017). However, this approach did not change the specialised care towards family medicine approach (Palamar and Gruzeva, 2018). In particular, the PHC facilities in urban areas function as Soviet policlinics with specialist-based care (Znatchkova, 2016; Vialkov *et al.*, 2017). Here care is delivered for adults by therapists and for children by paediatricians, working alongside other specialists (Ohanyan *et al*., 2015). This factor has adversely impacted family doctors’ ability to solve first-contact chronic health problems (Orynbassarova, 2015; Sh. Adilov, 2016). As for rural areas, due to the shortage of specialists, family doctors deliver NCD care (Mohir Ahmedov *et al.*, 2013). Some studies also discuss the roles nurses could play in CIS countries (Volodina, 2017, Mashpanina, 2017; Blake *et al*., 2019). Another weakness is that there is a lack of continuum of care for people with NCDs (Mashpanina, 2017; Beran *et al.*, 2019; Tolokonskaya *et al*., 2020).

Family doctors at PHC facilities are also overloaded with administrative and paperwork, which prevents them from allocating more time for patients’ counselling (Mukeeva, 2012). The appointment system, which was used in Soviet time, is no longer in place, and the flow of patients is unpredictable and not regulated (Hardison *et al*., 2007; Smith, 2011, Zhdan *et al*., 2017). Some of the CIS countries (Kazakhstan, Kyrgyzstan) have introduced mobile communication systems and e-cards for patients, but these have not reduced the workload of the family doctors, because the paper version was not discontinued (Zhdan *et al.*, 2017; Smiianov *et al.*, 2018). Kyrgyzstan is known as one of the first countries among CIS countries to introduce family medicine (Hardison *et al.*, 2007; Smith, 2011). However, attempts to integrate vertical services (tuberculosis care, endocrinology and oncology) in the PHC system in Kyrgyzstan and in other CIS countries resulted in increased workload of PHC (Ikeda *et al*., 2014; John Tayu Lee *et al*., 2015; Skordis-Worrall *et al.*, 2017; Bikbov *et al*., 2019).

The issue of quality of the services delivered at PHC in CIS countries was discussed in several studies (*Peabody et al.*, 2014; Sharman, 2014; Shinbolatova *et al.*, 2014; Orynbassarova, 2015; Peabody *et al.*, 2017). The studies conducted in Kazakhstan and Kyrgyzstan on CVD and hypertension confirmed that screening programmes and increasing awareness of patients about early detection of hypertension could contribute to the improvement of population’s health (Shinbolatova *et al.*, 2014; Kutlu *et al.*, 2014; Barbazza *et al*., 2019). In Ukraine and Kyrgyzstan, studies showed that outcome of the ambulance services was improved in the regions with the strong PHC system (Nasirdin Kyzy and Baatyrova, 2013; Voronenko and Shekera, 2013; Abanto 2018; Shekera, 2019). After 2010, Russia and Ukraine started reforms of the PHC system with patient-centric approach, by strengthening gatekeeping function and by introducing counselling for prevention NCD risk factors (Boitsov *et al.*, 2013a, 2013b; Peabody *et al.*, 2014; Gruzeva *et al.*, 2016b; Znatchkova, 2016; Volodina, 2017). In Uzbekistan, an evaluation of the quality of treatment of chronic heart failure at the PHC level find that the clinical protocols were very complicated to apply and follow by the family doctors (Ahmedov *et al*., 2013). Another finding from Tajikistan showed that the introduction of citizen report cards improved quality of care by defining problems, setting priorities, and monitoring healthcare providers (Bauhoff *et al.*, 2017). Another intervention inherited from soviet period is screening of population to detect chronic diseases, including NCDs, and to register them for continuous care at the PHC facilities (Boitsov *et al.*, 2013b; Balashova *et al.*, 2014).

To address the systemic issues such as excessive workload, shortage of human resources, poor quality of care, some CIS countries, Moldova (Codreanu *et al.,* 2012; Collins *et al.*, 2019; Collins *et al.*, 2020) and Kyrgyzstan (Jill Farrington, 2017) have piloted the WHO PEN protocol. The PEN pilot in Moldova was evaluated after two years of implementation and confirmed sustainable improvements in NCD risk factor control in primary health care conditioned to clinical training and support within two years (Collins *et al.*, 2020). In case of possible scaling up to a national level, this model could significantly reduce premature mortality from NCDs.

One of the studies in the Central Asian countries about gender gap of PHC services utilisation showed that children and women consume the overwhelming majority of PHC resources. Given the excess morbidity and mortality among men in the region it is required to redirect the PHC resources to address men’s health issues. This was also confirmed in a study done in Russia, which confirmed that the level of untreated hypertension was 28% higher among men (Petersen *et al.,* 2020). This issue could be addressed through specific outreach programmes at the PHC level, which was a basis for incentives to family doctors and nurses (Cashin *et al.*, 2002). As for the specific targeted activities to fight the NCDs, Russia started to establish a partnership with other CIS countries to address the NCD-related risk factors (Boitsov and Fleck, 2015).

## Discussion

In this review, a total of 96 documents were identified and analysed. These studies related to the primary healthcare system of the CIS countries and their work on NCD control, in particular hypertension and diabetes mellitus. Most of the studies came from Russia (33/96), Ukraine (18/96), and Kazakhstan (12/96). Turkmenistan was the only CIS country with no publications identified. All reviewed studies were analysed and clustered against WHO Health System Building Blocks. Most of the publications were about service delivery and human resources components, while few addressed information and research, and only one publication was related to medical products. As for the NCD disease groups, most studies focused on hypertension and much less on diabetes.

In general, the studies highlight the importance of PHC to better control and manage NCDs in CIS countries. However, in all CIS countries the PHC system is very diverse and still contains elements from the Soviet system, such as specialists working at PHC as general practitioners. There is no unique strategy specifically focused on strengthening PHC; thus, each of the countries is trying to find their own ways. The WHO PEN protocol could serve as a useful tool for the CIS countries to better integrate NCDs in primary health care, but only in Moldova and Kyrgyzstan (WHO-Europe, 2017; Collins *et al*., 2019) to date have piloted this protocol. As part of *leadership and governance block*, the CIS countries adopted WHO Global Action Plan to control NCDs, but there is no report on enforcement and results of this action plan. From a *health financing* point of view, the studies confirmed that CIS countries are spending much more for hospital care and very little for the PHC system (Oleszczyk *et al.*, 2012; Shvets, 2012; Schwarz *et al*., 2013). Donor interests to strengthen PHC in CIS countries are also very limited. This is also a reason of high (more than 40% of health expenditure) out-of-pocket expenditures in most of the CIS countries. In terms *of human resources* block, the education system is not adapted to train family physicians despite the importance of PHC in the CIS countries. In terms of *medical products*, only one study reported challenges with regard to availability and affordability of essential drugs at the PHC system. Only in Russia and Kazakhstan does family medicine exist as a scientific pillar (found from catalogue of scientific disciplines), while in other countries family medicine is not acknowledged as a scientific discipline. In terms of *service delivery*, major findings in this area show that PHC system is still disease-centric and delivered mostly by specialists instead of family doctors. Due to high administrative workload, family doctors do not have time for prevention activities, and in consequence, they mainly deal with the complications of diseases.

CIS countries are yet to implement true reforms oriented towards family medicine/general practice, due to the highly specialised care at PHC level. The Governments of CIS countries should develop a vision towards a stronger role of family doctors in NCD management and reconsider the role of specialists in the PHC system, either by retraining them as family doctors or as specialists required in rural areas. WHO should also take more proactive position by promoting adoption and implementation of the PEN protocol and offering best practices from other countries. Namely, WHO should support piloting of these strategies based on the demonstrated results and promote their adoption in targeted countries. A major gap in developing a global strategy and its implementation at national levels is the absence of steering and coaching mechanism at the WHO. WHO has developed strategies and policies as recommendations, but does not have any instruments to support countries in their adoption and implementation. The WHO country offices should play a bigger role in promoting the implementation of global strategies with closer steering and coaching mechanisms. WHO has programmes with specific focus on different elements of health systems strengthening. These programmes would have been redesigned with more focused approach to promote implementation of the concerned global strategies.

### Limitations of the review

The major limitation of the review was lack of publications on the NCD control at the primary healthcare level in the CIS countries, in particular published in English. Another limitation is that most of the studies and publications around PEN protocol implementation were done by WHO. The publications in Russian were more focused on the specific diseases, not on the NCD in general.

## Conclusion

The fact that only 96 studies were found over the last 10 years in the CIS countries related to NCDs and PHC system confirmed a limited interest to research and study in this area in CIS countries. Major reasons of this situation encompass a limited PHC reforms and lack of research programmes around PHC systems and NCDs. Development of strong PHC system is not adequately prioritised in the CIS countries, which resulted in low reputation and attractiveness of family medicine. Thus, there is limited development of family medicine as an academic and scientific specialty. The Governments of the CIS countries should revise their priorities towards strong PHC with patient- and prevention-centric approach, as PHC is the most affordable and effective type of care in the context of LMIC. As family medicine is a relatively new specialty, it requires better development from academic, clinical practice, and scientific perspectives.

## References

[ref1] Abanto V (2018) The Primary Health Care and practical ambulance service. Emergency Medicine, 12.

[ref2] Ahmedov M , Green J , Azimov R , Avezova G , Inakov S , Mamatkulov B (2013) Addressing the challenges of improving primary care quality in Uzbekistan: a qualitative study of chronic heart failure management. Health Policy Plan 28, 458–66. 22987825 10.1093/heapol/czs091

[ref3] Akhmedov R , Jarylkasynova NS (2010) General Medical Practice/Family Medicine formation experience in Bukhara State Medical Institute. Science About Health/Науки о здоровье, 7.

[ref4] Alikhanova KAT , Аsenova L , Такirova А , Omarkulov B (2017) Questions of training of general practitioners in priority areas of primary health care. Science-Review.ru Scientific Journal, 2.

[ref5] Alshamsan R , Lee JT , Rana S , Areabi H , Millett C (2017) Comparative health system performance in six middle-income countries: cross-sectional analysis using World Health Organization study of global ageing and health. Journal of the Royal Society of Medicine 110, 365–375. 28895493 10.1177/0141076817724599 PMC5987910

[ref6] Asadov D , Aripov T (2009) The quality of care in post-soviet Uzbekistan: are health reforms and international efforts succeeding? Public Health 123, 725–728. 19889431 10.1016/j.puhe.2009.09.013

[ref7] Avdeeva YL , Luchkevich V (2013) Modernization of prevention of chronic non-communicable diseases in the Primary Health Care system. Vrach-Doctor 11, 83–85.

[ref8] Babar ZU , Ramzan S , El-Dahiyat F , Tachmazidis I , Adebisi A , Hasan SS (2019) The availability, pricing, and affordability of essential diabetes medicines in 17 low-, middle-, and high-income countries. Frontiers in Pharmacology 10, 1375. 31824316 10.3389/fphar.2019.01375 PMC6880243

[ref9] Balabanova D , Roberts B , Richardson E , Haerpfer C , McKee M (2012) Health care reform in the former Soviet Union: beyond the transition. Health Services Research 47, 840–64. 22092004 10.1111/j.1475-6773.2011.01323.x PMC3419892

[ref10] Balashova ME , S GN , Karachurina GV (2014) The role of centers of general practice in prevention of diseases. Bulletin of Medical Internet Conferences, ISSN-2224-6150, 4, 4.

[ref11] Barbazza E , Yegeubayeva S , Akkazieva B , Tsoyi E , Zheleznyakov E , Tello JE (2019) Improving clinical practice in primary care for the prevention and control of noncommunicable diseases: a multi-actor approach to two regional pilot projects in Kazakhstan. Cardiovascular Diagnosis and Therapy 9, 129–139. 31143634 10.21037/cdt.2018.01.07 PMC6511679

[ref12] Barton SE (2011) Family Medicine – right choice for Kyrgyzstan. Vestnik Kyrgyzskoi Medicinskoi Akademii.

[ref13] Basu S , Millett C (2013) Social epidemiology of hypertension in middle-income countries: determinants of prevalence, diagnosis, treatment, and control in the WHO SAGE study. Hypertension 62, 18–26. 23670299 10.1161/HYPERTENSIONAHA.113.01374

[ref14] Bauhoff S , Rabinovich L , Mayer LA (2017) Developing citizen report cards for primary health care in low and middle-income countries: Results from cognitive interviews in rural Tajikistan. PLoS One 12, e0186745. 29065147 10.1371/journal.pone.0186745 PMC5655492

[ref15] Beran D , Perel P , Miranda JJ (2019) Forty years since Alma-Ata: do we need a new model for noncommunicable diseases? Journal of Global Health 9, 010316. 31217953 10.7189/jogh.09.010316 PMC6551545

[ref16] Bikbov MM , Fayzrakhmanov RR , Kazakbaeva GM , Zainullin RM , Arslangareeva II , Gilmanshin TR , Salavatova VF , Nikitin NA , Mukhamadieva SR , Yakupova DF , Khikmatullin RI , Zaynetdinov AF , Uzianbaeva YV , Aminev SK , Nuriev IF , Jonas JB (2019) Prevalence, awareness and control of diabetes in Russia: the Ural Eye and Medical Study on adults aged 40+ years. PLoS One 14, e0215636. 31009496 10.1371/journal.pone.0215636 PMC6476495

[ref17] Birn AE , Krementsov N (2018) ‘Socialising’ primary care? The Soviet Union, WHO and the 1978 Alma-Ata Conference. BMJ Global Health 3, e000992. 10.1136/bmjgh-2018-000992 PMC6242026 30498594

[ref18] Birtanov Y (2016) Kazakhstan gears up to launch social health insurance. Bulletin of World Health Organization 94, 792–793. 10.2471/BLT.16.031116 PMC5096350 27821881

[ref19] Blake C , Bohle LF , Rotaru C , Zarbailov N , Sava V , Secula F , Prytherch H , Curteanu A (2019) Quality of care for non-communicable diseases in the Republic of Moldova: a survey across primary health care facilities and pharmacies. BMC Health Services Research 19, 353. 31164125 10.1186/s12913-019-4180-4 PMC6547568

[ref20] Blanche Greene-Cramer AS , Lopes-Cardozo B , Husain F , Couture A , Bilukha O (2020) Noncommunicable disease burden among conflict-affected adults in Ukraine: a cross-sectional study of prevalence, risk factors, and effect of conflict on severity of disease and access to care. PLoS One 15 (4): e0231899. doi: 10.1371/journal.pone.0231899. 32315357 PMC7173772

[ref21] Boitsov SA , Gileva FA , Gulin AN , Ipatov PV , Kalinina AM , Linchak RM , Ponomareva EG (2013a) Improving the prevention of chronic non-communicable diseases in health care facilities. Preventive Medicine, 3–12.

[ref22] Boitsov SA , Fleck F (2015) Noncommunicable diseases: stepping up the fight. Bulletin of the World Health Organization 93, 9–10. 25558102 10.2471/BLT.15.030115 PMC4271693

[ref23] Boitsov SA , Kalinina AM , Ipatov PV (2013b) New clinical and organizational approaches to preventing cardiovascular diseases in the primary health care system. Therapeutic Archive 85, 8–13. 24137958

[ref24] Boitsov SA , Vylegzhanin SV (2015) Prevention of noncommunicable diseases in a local therapist’s practice: content, problems, solution ways, and prospects. Therapeutic Archive 87, 4–9. 10.17116/terarkh20158714-9 25823263

[ref25] Brimkulov NN (2015) Past, present and future of Family Medicine in Kyrgyzstan. Russian Family Doctor 19, 33–36.

[ref26] Cashin CE , Borowitz M , Zuess O (2002) The gender gap in primary health care resource utilization in Central Asia. Health Policy Plan, 17, 264–72. 12135992 10.1093/heapol/17.3.264

[ref27] Chizova IE , Oshchepkova E.V (2013) Results of the Federal (National) Project for prevention and treatment essential hypertension patients in Russia from 2002-2012 years. Vestnik Rossiskoi Akademii Medicinskih Nauk, 4–11. 10.15690/vramn.v68i2.542 23819322

[ref28] Codreanu I , Sali V , Gaibu S , Suveica L , Popa S , Perico N , Ene-Iordache B , Carminati S , Feehally J , Remuzzi G (2012) Prevalence of hypertension and diabetes and coexistence of chronic kidney disease and cardiovascular risk in the population of the republic of Moldova. International Journal on Hypertension 2012, 951734. 10.1155/2012/951734 PMC3515913 23251790

[ref29] Collins D , Ciobanu A , Laatikainen T , Curocichin G , Salaru V , Zatic T , Anisei A , Farrington J (2019) Protocol for the evaluation of a pilot implementation of essential interventions for the prevention of cardiovascular diseases in primary healthcare in the Republic of Moldova. BMJ Open 9, e025705. 10.1136/bmjopen-2018-025705 PMC6615880 31278091

[ref30] Collins D. , Inglin L , Laatikainen T , Ciobanu A , Curocichin G , Salaru V , Zatic T , Anisei A , Chiosa D , Munteanu M , Alexa Z , Farrington J (2020) Implementing a package of noncommunicable disease interventions in the Republic of Moldova: two-year follow-up data. Primary Health Care Research and Development 21, e39. 32993832 10.1017/S1463423620000420 PMC7576543

[ref31] Dzhakeli IV , Edzhibadze OI , Gerzmava O (2009) Management problems of improving the quality and efficiency of primary health care system of Georgia. Georgian Medical News, 96–99. 19644201

[ref32] Efremov DV (2011) About preventive work of the doctor working in primary health care. Problemy Sotsialnoi Gigieny, Zdravookhranenniia i Istorii Medicine 0869-866X, 30–32.

[ref33] Exworthy M (2008) The enduring legacy of Alma Ata: 30 years on. London Journal Primary Care (Abingdon) 1, 81–4. 10.1080/17571472.2008.11493214 PMC4212740 25949564

[ref34] Gazizov R. (2010) Historical milestones of Department of Therapy and Family medicine. Practical Medicine 135, 2.

[ref35] Gruzeva TS , V AD , Zamkevich VB (2016a) Harmful alcohol consumption: prevalence, trends, health burden, reduction strategy. Wiad Lek 69, 183–9. 27487531

[ref36] Gruzeva TS , P IM , Simyanov VA , Galienko L (2016b) Conceptual assumptions to create a system for preparation of healthcare human resources in Ukraine. Wiad Lek 69, 719–725. 28214802

[ref37] Gruzieva TS , Galiienko LI , Pelo IM , Omelchuk ST , Antonuk OY (2018) Health and lifestyle of students’ youth: status, problems and ways of solution. Wiad Lek 71, 1753–1758. 30737935

[ref38] Hardison C , Fonken P , Chew T , Smith B (2007) The emergence of family medicine in Kyrgyzstan. Family Medicine 39, 627–33. 17932795

[ref39] Hrytsko R , Furtak II , Parobets’ka IM (2014) Experience and problems of the primary health care centers’ specialists in the implementation of the integrated health system based on family medicine in Ukraine. Wiad Lek 67, 298–301. 25796853

[ref40] Ikeda N , Sapienza D , Guerrero R , Aekplakorn W , Naghavi M , Mokdad AH , Lozano R , Murray CJ , Lim SS (2014) Control of hypertension with medication: a comparative analysis of national surveys in 20 countries. Bulletin of the World Health Organization 92, 10–19C. 24391296 10.2471/BLT.13.121954 PMC3865548

[ref41] Jill Farrington, WEO (2017) Implementation of a package of essential noncommunicable (PEN) disease interventions in Kyrgyzstan: evaluation of effects and costs in Bishkek after one year.

[ref42] John Tayu Lee, FH , Pati S , Atun R , Millett C (2015). Impact of noncommunicable disease multimorbidity on healthcare utilization and out of pocket expenditures in middle-income countries: cross sectional analysis. PLOS One, 18. 10.1371/journal.pone.0127199 PMC4496037 26154083

[ref43] Kononov O , Klymenko LV , Batsiura GV , Matiukha LF , Protsiuk OV , Klymenko OV , Trishinska MA , Pogorila OI (2019) Retrospective analysis of the medical documentation of patients who applied to the ambulatory of general practice - family medicine. Wiad Lek 72, 938–941. 31175800

[ref44] Konysbaeva EU (2017) Effectiveness of narrow specialized care in the Primary Health Care Facislities. Vestnik Kazahskogo nationalnogo Universiteta 4, 321–324.

[ref45] Kasimova DAZ (2018) General medical practice (family medicine): development perspectives. Collection of Scientific Articles XLI International Scientific and Practical Conference. London, UK: European Research: Innovation in Science, Education and Technology.

[ref46] Kolesnyk P , Svab I (2013). Development of family medicine in Ukraine. European Journal on General Practice 19, 261–5. 10.3109/13814788.2013.807791 23971996

[ref47] Kotseva KAE (2019). Lifestyle and impact on cardiovascular risk factor control in coronary patients across 27 countries: results from the European Society of Cardiology. European Journal on Preventive Cardiology 26, 824–835. 10.1177/2047487318825350 30739508

[ref48] Krzton-Krolewiecka A , Svab I , Oleszczyk M , Seifert B , Smithson WH , Windak A (2013). The development of academic family medicine in central and eastern Europe since 1990. BMC Family Practice 14, 37. 23510461 10.1186/1471-2296-14-37 PMC3608318

[ref49] Kutlu R , Murataliev T , Memetoglu ME (2014) Management of coronary artery disease in Kyrgyzstan: a comparison with Turkey and Europe according to European Action on Primary and secondary prevention by intervention to reduce events III results. Turk Kardiyoloji Dernegi Arsivi 42, 545–52. 25362945 10.5543/tkda.2014.97254

[ref50] Lilit Khachatryan AB (2013) Performance assessment through pre- and post-training evaluation of continuing medical education courses in prevention and management of cardio-vascular diseases in primary health care facilities of Armenia. Journal Community Health 38(6), 1132–9. 10.1007/s10900-013-9724-7 23824876

[ref51] Luck J , Peabody JW , Demaria LM , Alvarado CS , Menon R (2014) Patient and provider perspectives on quality and health system effectiveness in a transition economy: evidence from Ukraine. Social Science & Medicine 114, 57–65. 24911509 10.1016/j.socscimed.2014.05.034

[ref52] Lysenko AA (2011) Diabetes type 2 and cardio-vascular diseases: collision of two global noncommunicable epidemics. Russian Medical Journal 19, 802–804.

[ref53] Martinez R , Lloyd-Sherlock P , Soliz P , Ebrahim S , Vega E , Ordunez P , McKee M (2020) Trends in premature avertable mortality from non-communicable diseases for 195 countries and territories, 1990-2017: a population-based study. Lancet Global Health 8, e511–e523. 32199120 10.1016/S2214-109X(20)30035-8

[ref54] Mashpanina T (2017) Role of nurses in family medicine as one of the way of development personalized medicine. Astrahan, Russia: Personificirovannaya medicina - vybor budushego.

[ref55] Matiukha LF , Medvedovska NV , Bukhanovska TM , Dikhtiarenko IY (2020) Sociological study results of self-assessment possibilities for self-realization among doctors of general practice - family medicine in Ukraine. Wiad Lek 73, 454–456. 32285812

[ref56] Mirzikashvili N , Baramidze L (2017) The role of primary health care in assessing and preventing health risk factors of adolescents in Georgia. Georgian Medical News, 53–57. 28480850

[ref57] Mohir Ahmedov RA , Mutalova Z , Huseynov S (2013) The HIT Uzbekistan. In Observatory E , editor, Health system in transition. WHO. 25689490

[ref58] Mukeeva ST (2012) Problems of family medicine in Kyrgyzstan. Vestnik Kyrgyzskoi Medicinskoi Akademii 1, 135–143.

[ref59] Nasirdin Kyzy OB , Baatyrova GM (2013) The ways of optimization the primary health care services. Vestnik Oshskogo Gosudarstvennogo Universiteta 4, 140–143.

[ref60] O’Malley HAL (2002) Scoping studies: towards a methodological framework. International Journal of Social Research Methodology, 19–32.

[ref61] Ohanyan M , Danielyan A , Hopayian K , Mash B (2015) International primary care snapshots: Armenia and South Africa. British Journal of General Practice 65, 308–9. 10.3399/bjgp15X685381 PMC4439815 26009520

[ref62] Oleszczyk M , Svab I , Seifert B , Krzton-Krolewiecka A , Windak A (2012) Family medicine in post-communist Europe needs a boost. Exploring the position of family medicine in healthcare systems of Central and Eastern Europe and Russia. BMC Family Practice 13, 15. 22409775 10.1186/1471-2296-13-15 PMC3368769

[ref63] Orynbassarova D (2015) Family medicine as a model of primary health services delivery: a pilot study in Almaty, Kazakhstan. Central Asian Journal of Global Health 4, 209. 29138716 10.5195/cajgh.2015.209 PMC5661198

[ref64] Palamar B , Gruzeva T (2019) The estimation of economic effectiveness of preventive measures of non-infectious diseases. Wiad Lek 72, 1532–1541. 32012505

[ref65] Palamar BI , Gruzeva TS (2018) Criteria of economic effectiveness of preventive measures of chronic non-infectious diseases. Wiad Lek 71, 897–906. 30099432

[ref66] Parfitt B (2009) Health reform: the human resource challenges for Central Asian Commonwealth of Independent States (CIS) countries. Collegian 16, 35–40. 19388425 10.1016/j.colegn.2009.01.002

[ref67] Peabody JW , Demaria L , Smith O , Hoth A , Dragoti E , Luck J (2017) Large-scale evaluation of quality of care in 6 countries of Eastern Europe and Central Asia using clinical performance and value vignettes. Global Health: Science and Practice 5, 412–429. 28963174 10.9745/GHSP-D-17-00044 PMC5620338

[ref68] Peabody JW , Luck J , Demaria L , Menon R (2014) Quality of care and health status in Ukraine. BMC Health Services Research 14, 446. 25269470 10.1186/1472-6963-14-446 PMC4263055

[ref69] Petersen J , Kontsevaya A , McKee M , Kudryavtsev AV , Malyutina S , Cook S , Leon DA (2020) Untreated hypertension in Russian 35-69 year olds - a cross-sectional study. PLoS One 15, e0233801. 32470073 10.1371/journal.pone.0233801 PMC7259637

[ref70] Polyanskaya I (2013) Creation of risk factors correction system for chronic noncommunicable diseases in the Kemerovo region. Medicine in Kuzbass, ISSN:1819-0901, 55–58.

[ref71] Rahmonov S.B. , R Z. Y. , Nozirov DJX , DjurVaev SHM (2012) Issues of prevention of non-communicable diseases in Tajikistan. Nauchno-practicheskii jurnal TIPPMK 3, 51–53.

[ref72] Rechel B , Roberts B , Richardson E , Shishkin S , Shkolnikov VM , Leon DA , Bobak M , Karanikolos M , McKee M (2013). Health and health systems in the Commonwealth of Independent States. Lancet 381, 1145–55. 23541055 10.1016/S0140-6736(12)62084-4

[ref73] Ryngach NO , Vlasyk LY (2018) Burden of premature mortality caused by four main non-communicable diseases in Ukraine. Wiad Lek 71, 728–732. 29783257

[ref74] Schwarz J , Wyss K , Gulyamova ZM , Sharipov S (2013) Out-of-pocket expenditures for primary health care in Tajikistan: a time-trend analysis. BMC Health Services Research 13, 103. 23505990 10.1186/1472-6963-13-103 PMC3614449

[ref75] Sh. Adilov ZA (2016) The prevalence of noninfectious diseases and the structure of the main types of noninfectious diseases European Research Journal 5, 105–106.

[ref76] Sharman A (2014) A New Paradigm of Primary Health Care in Kazakhstan: Personalized, Community-based, Standardized, and Technology-driven. Central Asian Journal of Global Health 3, 186. 29755891 10.5195/cajgh.2014.186 PMC5927735

[ref77] Sheiman I , Shishkin S , Shevsky V (2018) The evolving Semashko model of primary health care: the case of the Russian Federation. Risk Management and Healthcare Policy 11, 209–220. 30464661 10.2147/RMHP.S168399 PMC6220729

[ref78] Shekera OG (2019) The foundation of an effective health care system of ukraine - family medicine. Wiad Lek 72, 107–111. 30796873

[ref79] Shinbolatova A , Kulzhanov M , Aringazina A , Nurbakhyt A (2014) Screening of arterial hypertension in the Republic of Kazakhstan: advantages, disadvantages and ways of improving. Iranian Journal of Public Health 43, 1695–701. 26171363 PMC4499092

[ref80] Shvets RI (2012) Problems of defining cost of the Family Medicine. Problemy Economiki, Harkov 2, 3–6.

[ref81] Skordis-Worrall J , Round J , Arnold M , Abdraimova A , Akkazieva B , Beran D (2017) Addressing the double-burden of diabetes and tuberculosis: lessons from Kyrgyzstan. Global Health 13, 16. 28298226 10.1186/s12992-017-0239-3 PMC5353796

[ref82] Smiianov VA , Dryha NO , Smiianova OI , Obodyak VK , Zudina TO (2018) Development of informational-communicative system, created to improve medical help for family medicine doctors. Wiad Lek 71, 331–334. 29786581

[ref83] Smith, B. (2011) *Family medicine - is it right choice for Kyrgyzstan?*

[ref84] Suhrcke, M. , Rechel, B. & Michaud, C. (2005) Development assistance for health in central and eastern European Region. Bulletin of the World Health Organization 83, 920–7. 16462984 PMC2626498

[ref85] Tolokonskaya N , CS, Gorenkov R , Agafonov B (2020) Role of Family Medicine in the implementation of new strategies and possibilities of health care within P4 medicine model. Therapist ISSN:2075-0277, 13–20.

[ref86] Verulava T , Jorbenadze R , Barkalaia T (2017) Introduction of Universal Health Program in Georgia: problems and perspectives. Georgian Medical News, 116–120. 28252441

[ref87] Vialkov AI , Skvirskaia GP , Son IM , Senenko AS , Kupeieva IA , Rosanov VB , Leonov SA , Bilalov FK , Gajeva AV , Evdakov VA , Kravtchenko NA (2017) The Actual Approaches to Reformation of Medical Organizations Providing Out-Patient Care to Population. Problemy sotsial’noi gigieny, zdravookhraneniia i istorii meditsiny 25, 216–220. 10.1016/0869-866X-2017-25-4-216-220 29510010

[ref88] Volodina AK (2017) Improvement of management of patients flow in Primary Health Care. Conference materials: Problems of effective governance - internal and external factors of dinamic development of Russia. Voronej, Russia: Nauchnaya Kniga.

[ref89] Voronenko YV , Shekera OG (2013) Family Medicine in the lead of the health sector reforms in Ukraine. Vestnik KSMA, 6.

[ref90] WHO-Europe RO (2017) *Workshop on implementation of a package of essential noncommunicable (PEN) disease interventions for primary health care in Eastern Europe and central Asia*.

[ref91] WHO-Moldova CO (2018) *Tackling noncommunicable diseases in the Republic of Moldova* [Online]. [Accessed].

[ref92] WHO-Ukraine CO (2018) *Tackling noncommunciable diseases in Ukraine*.

[ref93] WHO (2008). *2008-2013 Action Plan for the Global Strategy for the Prevention and Control of Noncommunicable Diseases*, *WHO web page* , WHO.

[ref94] WHO (2010). *Health system building blocks* [Online]. https://extranet.who.int/nhptool/BuildingBlock.aspx. [Accessed].

[ref95] WHO (2014) *NCD country profiles* [Online]. https://www.who.int/nmh/publications/ncd-profiles-2014/en/. [Accessed].

[ref96] WHO (2022) *Key facts on noncommunicable diseases* [Online]. https://www.who.int/news-room/fact-sheets/detail/noncommunicable-diseases. [Accessed].

[ref97] Zarbailov N , Wilm S , Tandeter H , Carelli F , Brekke M (2017) Strengthening general practice/family medicine in Europe-advice from professionals from 30 European countries. BMC Family Practice 18, 80. 28830385 10.1186/s12875-017-0653-x PMC5568085

[ref98] Zhdan VM , Holovanova IA , Filatova VL , Khorosh MV (2017) Medical evaluation of efficiency of optimized models for early detection and primary prevention of cardiovascular diseases. Wiad Lek 70, 433–438. 28711883

[ref99] Znatchkova EA (2016) On the issue of improving primary health care for adult population in Moscow. Problemy Sotsialnoi Gigieny Zdravookhranenniia i Istorii Mediciny 24, 98–101. 29553209

